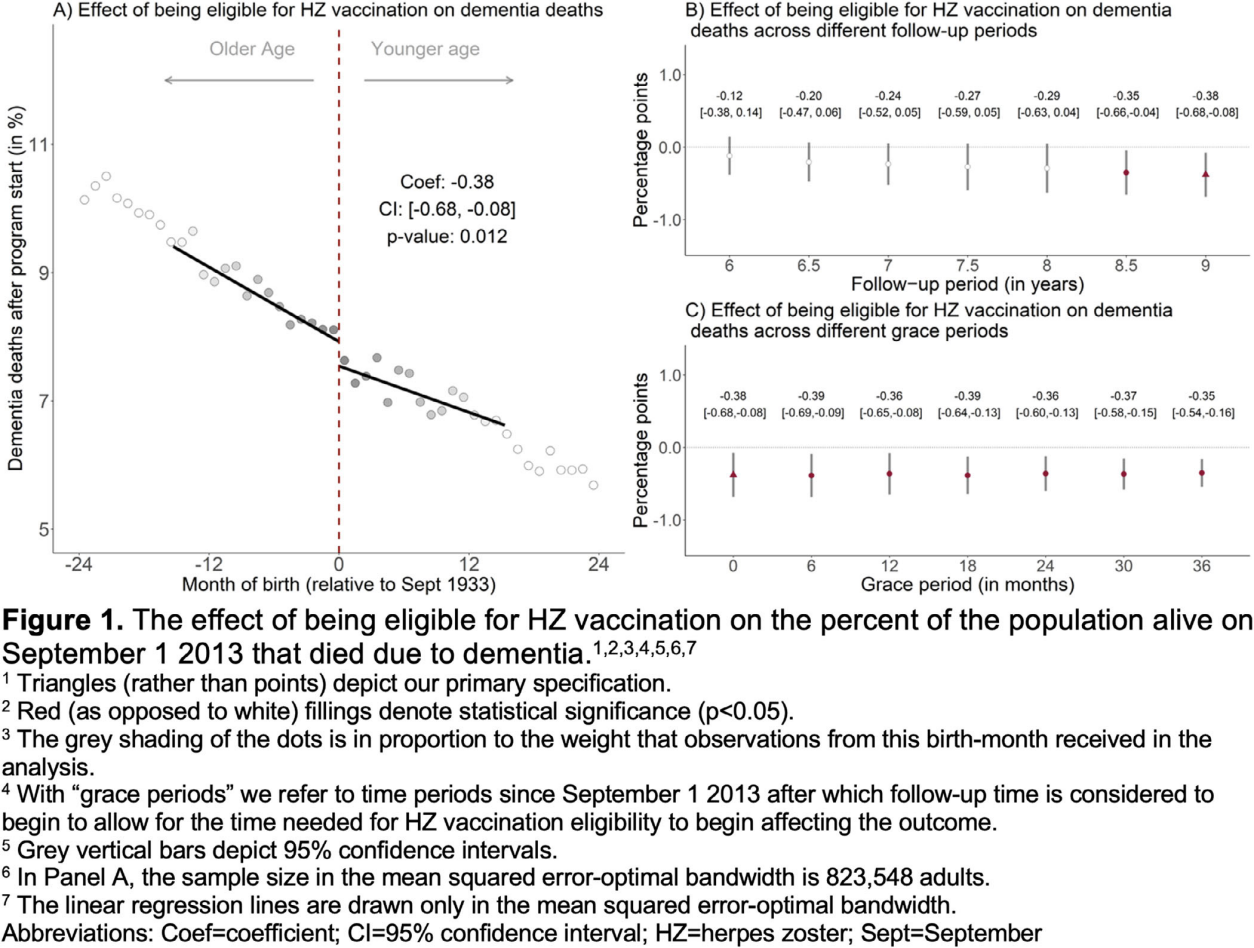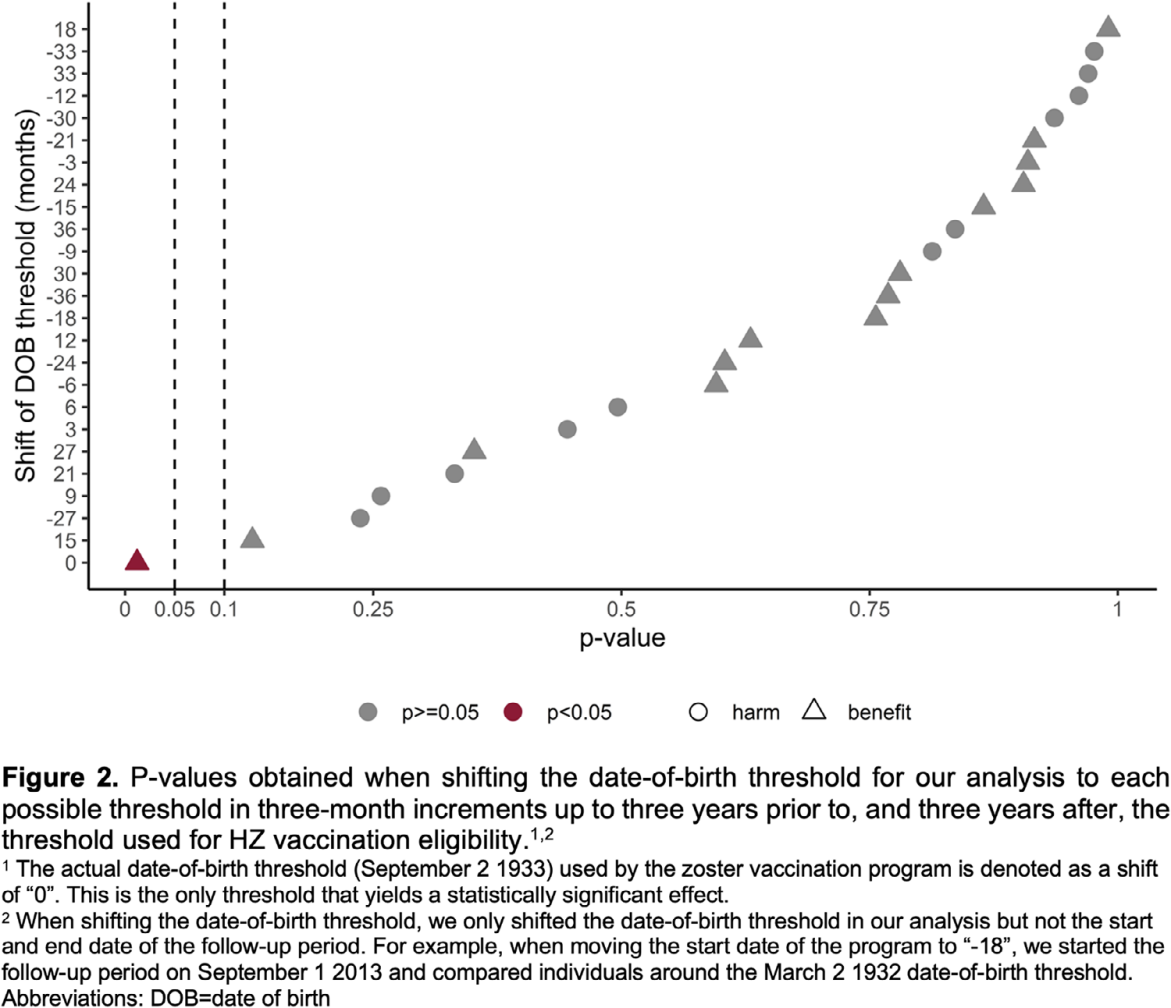# The effect of herpes zoster vaccination on the occurrence of deaths due to dementia in England and Wales

**DOI:** 10.1002/alz.088262

**Published:** 2025-01-09

**Authors:** Felix Michalik, Min Xie, Markus Eyting, Simon Hess, Seunghun Chung, Pascal Geldsetzer

**Affiliations:** ^1^ Stanford University, Stanford, CA USA; ^2^ Heidelberg University, Heidelberg Germany; ^3^ Heidelberg Institute of Global Health, Heidelberg, Baden‐Württemberg Germany; ^4^ Johannes Gutenberg University Mainz, Mainz, Rhineland‐Palatinate Germany; ^5^ University of Vienna, Vienna, Vienna Austria; ^6^ Chan Zuckerberg Biohub, San Francisco, CA USA

## Abstract

**Background:**

Using a unique natural randomization, we have recently provided evidence from Welsh electronic health record data that herpes zoster (HZ) vaccination caused a reduction in new dementia diagnoses over a seven‐year period. This study aimed to determine whether eligibility for HZ vaccination also caused a reduction in deaths due to dementia in England and Wales over a nine‐year follow‐up period.

**Methods:**

Adults who had their 80^th^ birthday shortly before September 1 2013 were ineligible for free HZ vaccination and remained ineligible for life, whereas those who had their 80^th^ birthday shortly after September 1 2013 (i.e., born on or after September 2 1933) were eligible for one year. This date‐of‐birth threshold generated birth cohorts who are likely exchangeable in observed and unobserved characteristics except for a small difference in age and a large difference in HZ vaccination uptake. We used country‐wide data from death certificates in England and Wales on underlying causes of death from September 1 2004 to August 31 2022 by ICD‐10 code and month of birth. Our analysis compared the percentage of the population with a death due to dementia among the month‐of‐birth cohorts around the September 2 1933 eligibility threshold using a regression discontinuity design.

**Results:**

The study population included 5,077,426 adults born between September 1 1925 and August 31 1941 who were alive at the start of the HZ vaccination program. We estimated that over a nine‐year follow‐up period, eligibility for HZ vaccination reduced the percentage of the population with a death due to dementia by 0.38 (95% CI: 0.08 to 0.68, p = 0.012) percentage points, corresponding to a relative reduction of 4.8%. As in our prior analysis, this effect was stronger among women (‐0.62 [95% CI: ‐1.06 to ‐0.19] percentage points, p = 0.004) than among men (‐0.11 [95% CI: ‐0.51 to 0.28] percentage points, p = 0.574).

**Conclusions:**

Our findings indicate that HZ vaccination reduced cognitive decline at a fairly advanced stage of the dementia disease process because most individuals whose underlying cause of death was dementia during our nine‐year follow‐up period were likely already living with dementia at the start of the HZ vaccination program.